# Pericardial effusion requiring intervention in patients undergoing leadless pacemaker implantation: A real-world analysis from the National Inpatient Sample database

**DOI:** 10.1016/j.hroo.2024.02.004

**Published:** 2024-02-19

**Authors:** Muhammad Zia Khan, Yasar Sattar, Waleed Alruwaili, Sameh Nassar, Mohamed Alhajji, Bandar Alyami, Amanda T. Nguyen, Joseph Neely, Zain Ul Abideen Asad, Siddharth Agarwal, Sameer Raina, Sudarshan Balla, Bao Nguyen, Dali Fan, Douglas Darden, Muhammad Bilal Munir

**Affiliations:** ∗ Division of Cardiology, West Virginia University Heart and Vascular Institute, Morgantown, West Virginia; † Section of Electrophysiology, Division of Cardiology, University of California Davis, Sacramento, California; ‡ Division of Cardiology, University of Oklahoma, Oklahoma City, Oklahoma; § Division of Cardiology, Stanford University, Stanford, California; || Division of Cardiology, Kansas City Heart Rhythm Institute, Overland Park, Kansas

**Keywords:** Leadless pacemakers, Pericardial effusion, Predictors, Outcomes

## Abstract

**Background:**

Pericardial effusion requiring percutaneous or surgical-based intervention remains an important complication of a leadless pacemaker implantation.

**Objective:**

The study sought to determine real-world prevalence, risk factors, and associated outcomes of pericardial effusion requiring intervention in leadless pacemaker implantations.

**Methods:**

The National Inpatient Sample and International Classification of Diseases–Tenth Revision codes were used to identify patients who underwent leadless pacemaker implantations during the years 2016 to 2020. The outcomes assessed in our study included prevalence of pericardial effusion requiring intervention, other procedural complications, and in-hospital outcomes. Predictors of pericardial effusion were also analyzed.

**Results:**

Pericardial effusion requiring intervention occurred in a total of 325 (1.1%) leadless pacemaker implantations. Patient-level characteristics that predicted development of a serious pericardial effusion included >75 years of age (odds ratio [OR] 1.38, 95% confidence interval [CI] 1.08–1.75), female sex (OR 2.03, 95% CI 1.62–2.55), coagulopathy (OR 1.50, 95% CI 1.12–1.99), chronic pulmonary disease (OR 1.36, 95% CI 1.07–1.74), chronic kidney disease (OR 1.53, 95% CI 1.22–1.94), and connective tissue disorders (OR 2.98, 95% CI 2.02–4.39). Pericardial effusion requiring intervention was independently associated with mortality (OR 5.66, 95% CI 4.24–7.56), prolonged length of stay (OR 1.36, 95% CI 1.07–1.73), and increased cost of hospitalization (OR 2.49, 95% CI 1.92–3.21) after leadless pacemaker implantation.

**Conclusion:**

In a large, contemporary, real-world cohort of leadless pacemaker implantations in the United States, the prevalence of pericardial effusion requiring intervention was 1.1%. Certain important patient-level characteristics predicted development of a significant pericardial effusion, and such effusions were associated with adverse outcomes after leadless pacemaker implantations.

## Graphical abstract

**Figure:**
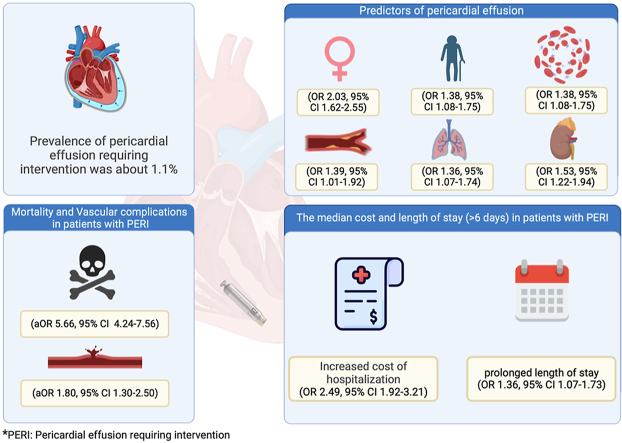

**Key Findings**

▪ Prevalence of pericardial effusion requiring intervention after a leadless pacemaker implantation was 1.1%.
▪ Advanced age, female sex, chronic pulmonary disease, chronic kidney disease, and connective tissue disorders predicted development of serious pericardial effusion after leadless pacemaker implantation.
▪ Significant pericardial effusion was associated with mortality, increased length of stay, and higher cost of hospitalization after leadless pacemaker implantation.

## Introduction

Leadless pacemaker is an important modality designed to manage bradyarrhythmias in selected patients who need pacing support. These devices provide durable pacing and are not associated with lead- or pocket-related complications occasionally witnessed with a conventional transvenous pacing system.^1^ Pericardial effusion requiring intervention (percutaneous or surgical) remains the most dreaded complication of leadless pacemaker implantation. The incidence of serious pericardial effusion in the landmark Micra Transcatheter Pacing Study was 1.6%.^2^ More recently, the LEADLESS II-Phase 2 ( Percutaneous Implantation of an Entirely Intracardiac Leadless Pacemaker) trial evaluating the efficacy and safety of the Aveir leadless pacemaker system (Abbott) showed the incidence of serious pericardial effusion to be about 1.5%.^3^ There is a paucity of real-world data on the prevalence, risk factors, and associated outcomes of pericardial effusion requiring either percutaneous or surgical drainage after leadless pacemaker implantation in the United States. We aimed to study these parameters from a large and nationally representative sample of the U.S. population.

## Methods

### Data source

Data from National Inpatient Sample (NIS) were used for the purpose of our current study. We analyzed the NIS database from years 2016 to 2020 for leadless pacemaker device implantations. On April 6, 2016, the Food and Drug Administration (FDA) approved the first leadless pacemaker device, the Medtronic Micra.^4^ The NIS is a large hospital based administrative database which was made possible by a federal-state-industry partnership sponsored by the Agency for Healthcare Research and Quality. The NIS can be used for computing national estimates of healthcare utilization, costs, and outcomes. The NIS provides discharge weights that are used for estimation of disease and procedure trends nationally. The data are de-identified; therefore, the need for informed consent and Institutional Review Board approval is waived.^5^ The NIS adheres to the 2013 Declaration of Helsinki for the conduct of human research.

### Study population

Leadless pacemaker device implantations were identified using International Classification of Diseases–Tenth Revision–Clinical Modification (ICD-10-CM) code 02HK3NZ. Patients younger than 18 years of age and those with missing demographic data were excluded. The study sample was stratified into 2 groups: no pericardial effusion after leadless pacemaker implantation and pericardial effusion requiring intervention (percutaneous or surgical) after leadless pacemaker implantation. For percutaneous drainage, ICD-10-CM codes 0W9D30Z and 0W9D3ZZ were used. For the open cardiac surgery–based intervention, ICD-10-CM codes 0W9D00Z and 0W9D0ZZ were used. These codes have been used in earlier studies for the extraction of patients who experienced pericardial effusion requiring intervention from the administrative datasets.^6^

Baseline characteristics, other procedural complications, and inpatient outcomes including mortality (reported as a distinct categorical variable in the dataset), length of stay, and hospitalization costs were compared between the 2 groups. We also analyzed the patient-level predictors of pericardial effusion requiring intervention after leadless pacemaker implantation. Independent associations of pericardial effusion requiring intervention (vs not) with important outcomes of mortality, vascular complications, prolonged length of stay (defined as >6 days), and increased hospitalization costs (defined as median cost >$34,098) were also analyzed. For computing hospitalization costs, the cost-to-charge ratio files based on Centers for Medicare and Medicaid Services reimbursement and provided by the Healthcare Cost and Utilization Project were applied to the total hospital charges.

### Statistical analysis

Descriptive statistics are presented as frequency and percentage for categorical variables and as median and interquartile range for continuous variables. Baseline characteristics were compared using a Pearson chi-square test and Fisher exact test for categorical variables and the Kruskal-Wallis H test for continuous variables. For crude comparison of procedural complications and in-hospital outcomes among the study groups, the Pearson chi-square test was used.

Logistic regression was performed to estimate odds ratios (ORs) with 95% confidence intervals (CIs) to determine patient-level predictors of pericardial effusion requiring percutaneous drainage or open cardiac surgery–based intervention after leadless pacemaker implantation. A forward stepwise entry model was used for this purpose. Initially, all variables, which were significantly associated with pericardial effusion with a *P* value of <.05 in univariable analysis, were entered into the model from the baseline table. Subsequently, only those variables were retained in the model that were associated with pericardial effusion with a *P* value of <.10 during forward entry. For the assessment of independent association of pericardial effusion with outcomes of mortality, vascular complications, prolonged length of stay, and increased hospitalization costs, a single-step multivariable logistic regression model was used. Age, sex, race/ethnicity, income, insurance status, and selected Elixhauser comorbidities were used for adjusted analysis. All these covariates were identified based on prior literature, bivariate analysis, and the authors’ best clinical judgment.^7^^,^^8^ A *P* value of <.05 was considered statistically significant. All statistical analyses were performed using SPSS version 26 (IBM Corporation) and R version 3.6 (R Foundation for Statistical Computing). Because of the complex survey design of the NIS, sample weights, strata, and clusters were applied to raw data to generate national estimates.^5^

## Results

A total of 29,005 leadless pacemakers were analyzed in our study after excluding for missing demographics. Of these, approximately 325 (1.1%) implantations were complicated by a serious pericardial effusion requiring percutaneous or surgical-based drainage. Baseline characteristics of the study population are shown in Table 1. The prevalence of pericardial effusion requiring intervention (vs not) were greater in patients with advanced age (82 years vs 77 years, *P <* .01) and women (60% vs 44.5%, *P <* .01). Comorbidities such as chronic pulmonary disease (29.2% vs 24.3%, *P <* .01), connective tissue disorders (9.2% vs 2.9%, *P <* .01), coagulopathy (18.5% vs 13%, *P <* .01), and chronic kidney disease (46.2% vs 40.8%, *P <* .01) were more prevalent in leadless pacemaker implantations complicated by pericardial effusion requiring intervention.

**Table 1:** Baseline characteristics of the study population

Variable	No pericardial effusion	Pericardial effusion requiring intervention	*P* value
Patients	28,680 (98.9)	325 (1.1)	
Age, y	77 (69–85)	82 (72–88)	<.01
Women	12,765 (44.5)	195 (60.0)	<.01
Age <65 y	4915 (17.1)	30 (9.2)	<.01
Age 65–74 y	6395 (22.3)	80 (24.6)	
Age ≥75 y	17,370 (60.6)	215 (66.2)	
Race/ethnicity			
White	21,320 (76.4)	245 (77.8)	<.01
Black	2760 (9.9)	30 (9.5)	
Hispanic	2065 (7.4)	25 (7.9)	
Asian or Pacific Islander	880 (3.2)	10 (3.2)	
Native American	105 (0.4)	5 (1.6)	
Other	760 (2.7)	0 (0.0)	
Comorbidities			
Deficiency anemia	1645 (5.7)	15 (4.6)	.38
Congestive heart failure	14,995 (52.3)	160 (49.2)	.27
Connective tissue disorders	825 (2.9)	30 (9.2)	<.01
Chronic pulmonary disease	6975 (24.3)	95 (29.2)	.04
Cerebrovascular disorders	3035 (10.6)	55 (16.9)	<.01
Coagulopathy	3730 (13.0)	60 (18.5)	<.01
Coronary artery disease	12,515 (43.6)	125 (38.5)	.06
Diabetes mellitus	2820 (9.8)	15 (4.6)	<.01
Hypertension	24,205 (84.4)	240 (73.8)	<.01
Major depression	2845 (9.90%	15 (4.60%	<.01
Hypothyroidism	5770 (20.1)	30 (9.2)	<.01
Liver disease	1780 (6.2)	20 (6.2)	.96
Obesity	5155 (18.0)	25 (7.7)	<.01
Peripheral vascular disorders	2920 (10.2)	45 (13.8)	.03
Chronic kidney disease	11,695 (40.8)	150 (46.2)	.05
Pathological weight loss	2965 (10.3)	60 (18.5)	<.01
Hospital location			
Rural	660 (2.3)	5 (1.5)	<.01
Urban nonteaching	4040 (14.1)	65 (20.0)	
Urban teaching	23,980 (83.6)	255 (78.5)	
Bed size of the hospital			
Small	2900 (10.1)	60 (18.5)	<.01
Medium	6930 (24.2)	50 (15.4)	
Large	18,850 (65.7)	215 (66.2)	
Payee			
Medicare	23,465 (81.9)	260 (80.0)	.18
Medicaid	1485 (5.2)	25 (7.7)	
Private insurance	2900 (10.1)	35 (10.8)	
Self-pay	270 (0.9)	0 (0.0)	
No charge	30 (0.1)	0 (0.0)	
Other	490 (1.7)	5 (1.5)	
Median income			
0–25	7235 (25.6)	80 (25.0)	.25
25–50	7395 (26.1)	95 (29.7)	
50–75	6940 (24.5)	65 (20.3)	
75–100	6725 (23.8)	80 (25.0)	

Values are n (%) or mean ± SD.

For n <10, the absolute numbers are not reported as per Healthcare Cost and Utilization Project recommendations.

Other important procedure-related complications and in-hospital outcomes after leadless pacemaker implantation and stratified on the basis of pericardial effusion requiring intervention vs not are shown in Tables 2 and 3, respectively. The prevalence of any peripheral vascular complication was higher in patients with a course complicated by a serious pericardial effusion vs not (13.8% vs 7.5%, *P <* .01). No difference in the rate of acute kidney injury was demonstrated in both groups (29.2% vs 30.9%, *P =* .5). In-hospital deaths were more prevalent in leadless pacemaker implantations complicated by pericardial effusion requiring intervention (21.5% vs 4.8%, *P <* .01). Leadless pacemaker implantations complicated by pericardial effusion requiring intervention were also noted to have longer length of stay (8 days vs 6 days, *P <* .01) and increased cost of hospitalization ($54,142 vs $33,913, *P <* .01).

**Table 2:** Other leadless pacemaker procedure related complications

Variable	No pericardial effusion	Pericardial effusion requiring intervention	*P* value
Patients	28,680 (98.9)	325 (1.1)	
Any peripheral vascular complication∗	2165 (7.5)	45 (13.8)	<.01
AV fistula	65 (0.2)	10 (3.1)	<.01
Pseudoaneurysm	230 (0.8)	10 (3.1)	<.01
Hematoma	450 (1.6)	10 (3.1)	.03
Retroperitoneal bleeding	100 (0.3)	<10 (1.5)	<.01
Venous thromboembolism	1405 (4.9)	20 (6.2)	.30
Acute kidney injury	8870 (30.9)	95 (29.2)	.5

Values are n (%). For n < 10, the absolute numbers are not reported as per Healthcare Cost and Utilization Project recommendations.

AV = arteriovenous.

∗ Defined as a composite of AV fistula, pseudoaneurysm, hematoma, retroperitoneal bleeding, and venous thromboembolism.

**Table 3:** Hospital outcomes in leadless pacemaker recipients

Variable	No pericardial effusion	Pericardial effusion requiring intervention	*P* value
Patients	28,680 (98.9)	325 (1.1)	
Died at discharge	1370 (4.8)	70 (21.5)	<.01
Discharge disposition			
Home/routine/self-care	18,155 (66.5)	150 (58.8)	<.01
Nonhome discharges	9155 (33.5)	105 (41.2)	
Resource utilization			
Length of stay, d	6 (3–11)	8 (4–17)	<.01
Cost of hospitalization, $	33,913 (23,306–55,512)	54,142 (33,490–95,022)	<.01

Values are n (%) or median (interquartile range).

**Figure 1:**
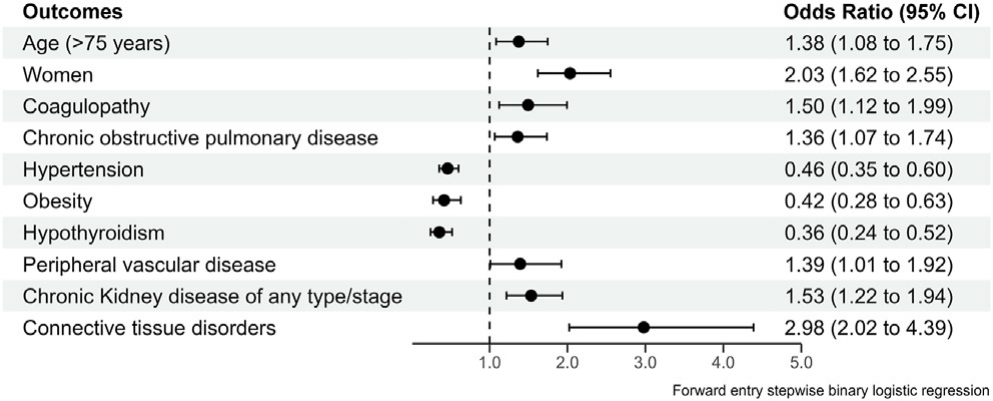
Patient-level predictors of pericardial effusion requiring intervention after leadless pacemaker implantation. CI = confidence interval.

Patient-level characteristics that predicted the development of pericardial effusion requiring intervention are shown in Figure 1. Age >75 years (OR 1.38, 95% CI 1.08–1.75), female sex (OR 2.03, 95% CI 1.62–2.55), coagulopathy (OR 1.50, 95% CI 1.12–1.99), chronic pulmonary disease (OR 1.36, 95% CI 1.07–1.74), chronic kidney disease (OR 1.53, 95% CI 1.22–1.94), and connective tissue disorders (OR 2.98, 95% CI 2.02–4.39) were associated with a high likelihood of pericardial effusion requiring intervention. Hypertension (OR 0.46, 95% CI 0.35–0.60), obesity (OR 0.42, 95% CI 0.28–0.63), and hypothyroidism (OR 0.36, 95% CI 0.24–0.52) were associated with a low likelihood of pericardial effusion requiring intervention after leadless pacemaker implantation.

**Figure 2:**
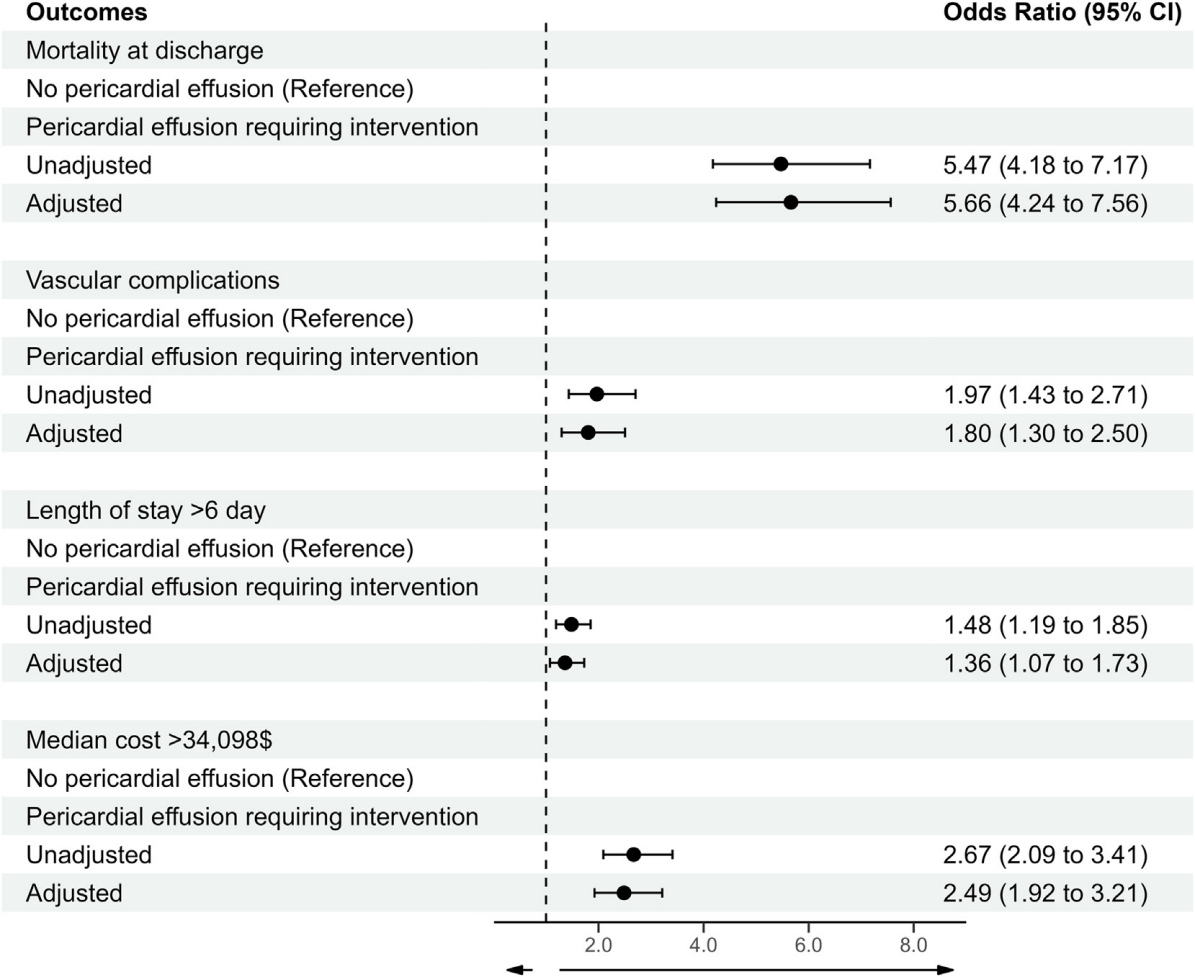
Adjusted association of pericardial effusion requiring intervention with outcomes of mortality, any vascular complication, prolonged length of stay, and increased hospitalization costs. CI = confidence interval.

To assess the independent association of the complication of pericardial effusion requiring intervention with other in-hospital outcomes, we constructed multivariable models adjusting for potential confounders, which are shown in Figure 2. After adjustment, pericardial effusion requiring intervention was associated with in-hospital mortality (OR 5.66, 95% CI 4.24–7.56), any vascular complication (OR 1.80, 95% CI 1.30–2.50), prolonged length of stay (OR 1.36, 95% CI 1.07–1.73), and increased cost of hospitalization (OR 2.49, 95% CI 1.92–3.21).

## Discussion

In this contemporary, real-world study of leadless pacemaker implantations from the United States, we report several important findings: (1) the prevalence of pericardial effusion requiring percutaneous or surgical drainage was 1.1%; (2) certain patient characteristics such as advanced age, female sex, coagulopathy, chronic pulmonary disease, chronic kidney disease, and connective tissue disorders were associated with an increased risk, while hypertension, obesity, and hypothyroidism were associated with a decreased risk of pericardial effusion requiring intervention after leadless pacemaker implantation; and (3) pericardial effusion requiring intervention was independently associated with other important outcomes of mortality, any vascular complication, prolonged length of stay, and increased hospitalization cost after leadless pacemaker implantation.

Before the advent of leadless pacemakers, patients with symptomatic bradycardia were managed exclusively with a transvenous pacing system consisting of a pulse generator, which was placed in a surgically created pocket and leads implanted inside the heart through the veins in the upper torso. Approximately 1 in 8 patients experienced an early complication after placement of these devices, and these complications are usually related to the pocket or the leads.^1^ Leadless pacemakers are designed to mitigate some of the complications associated with conventional transvenous pacing systems and to extend the pacing modality in management of those patients in whom the conventional pacing system is undesirable (such as patients with recurrent infection).^2^^,^^3^ Significant pericardial effusion remains the most feared complication of leadless pacemaker implantation. The pivotal Micra Transcatheter Pacing Study evaluating the efficacy and safety of Medtronic Micra leadless pacing system showed that approximately 1.6% of such implantations were complicated by a serious pericardial effusion.^2^ Additionally, the incidence of a serious pericardial effusion in the most recent LEADLESS II-Phase 2 trial (Aveir system) was 1.5%, and most of these effusions were managed with an open surgical drainage.^3^ In our real-world and contemporary cohort of U.S. practice, the prevalence of pericardial effusion requiring percutaneous or surgical drainage after the leadless pacemaker implantation was 1.1%, which is lower when compared with controlled trials indicating a broader safety of such implantations across various patient groups. In another study of more than 1800 leadless pacemaker implantations from the Micra postapproval registry, El-Chami and colleagues^9^ reported an even lower rate of cardiac perforation of 0.77% after such implantations. Piccini and colleagues^10^ analyzed more than 2800 leadless pacemaker implantations from the Micra global trials and found the rate of pericardial effusion to be 1.1%. Similar to our study, they found advanced age, female sex, and chronic pulmonary disease to be associated with pericardial effusion after leadless pacemaker implantation.^10^ In a systematic review enrolling studies on conventional and leadless pacemakers, Vamos and colleagues^11^ reported cardiac perforation rate in excess of 1.5% after leadless pacemaker implantations, which was higher than what was observed with conventional pacemakers.

Our study also demonstrated that advanced age, women, coagulopathy, chronic pulmonary disease, chronic kidney disease, and connective tissue disorders were associated with an increased risk of pericardial effusion requiring intervention after leadless pacemaker implantation. The Micra Transcatheter Pacing Study also noted an increased risk of serious pericardial effusion after Micra implantation in the elderly, women, and patients with history of chronic lung disease, although the magnitude of such associations was not reported in that study.^2^ It is plausible that older patients and women have a smaller right ventricular cavity with a tendency toward apical displacement of leadless pacemaker at the time of implantation, and the myocardial tissue is especially thinner in that region, thus enhancing the risk of rupture and resulting pericardial effusion.^12^ Indeed, all the cardiac perforations and resultant pericardial effusions witnessed in the LEADLESS II-Phase 2 Aveir trial were associated with the apical placement of the device.^3^ Connective tissue disorders were strongly associated with the risk of pericardial effusion requiring intervention in our study, which is likely related to long-term steroid use in these patients. In fact, in a study of more than 4000 conventional permanent pacemaker implantations, Mahapatra and colleagues^13^ showed that steroid use was strongly associated with the risk of cardiac perforation and pericardial effusion (hazard ratio 4.1, 95% CI 1.1–10). Our study also showed that hypertension and hypothyroidism were protective against the development of serious pericardial effusion after leadless pacemaker implantation. Both of these clinical entities are associated with myocardial and pericardial fibrosis, and that may protect against any untoward cardiac perforation and pericardial effusion.^14^^,^^15^ It is worth pointing out that our data are not equipped to analyze the underlying mechanisms of such associations and should therefore be the subject of future investigations with the goal of making leadless pacemaker implantations more safer.

The absolute difference in mortality among leadless pacemaker patients with and without significant pericardial effusion was striking (21.5% vs 4.8%, *P <* .01). Leadless pacemaker implantations complicated by pericardial effusion requiring intervention were also noted to have longer length of stay (8 days vs 6 days, *P <* .01) and increased cost of hospitalization ($54,142 vs $33,913, *P <* .01) in crude analysis. These differences exist despite adjusting for confounders, suggesting that serious pericardial effusion remains an important contributor to poor patient outcomes and increased resource utilization after leadless pacemaker implantation. Use of contrast and steep fluoroscopic angles and occasional utilization of imaging modalities such as echocardiography during leadless pacemaker implantation can further improve the safety of such procedures, as they aid in avoiding apical right ventricular placement and should be considered especially in high-risk patient subgroups.^16^

### Limitations

The results of our current study should be interpreted in the context of following limitations. First, the NIS relies on ICD codes for disease and procedure identification, which may be subject to errors; however, the NIS uses a rigorous data quality control program to minimize miscoding and ensures integrity of data.^5^ Second, long-term outcomes cannot be ascertained from the present dataset, as the NIS includes index admission data only. Third, there are no data available on procedural steps involved with leadless pacemaker implantation such as utilization of contrast, intraprocedural imaging, and operator experience. Fourth, the NIS only caters to inpatient admissions and does not provide information on outpatient encounters. Fifth, the NIS is not equipped to delineate the granular mechanisms of pericardial effusion etiologies associated with implantation of a leadless pacemaker. Sixth, our analysis is limited primarily to the Micra leadless pacemaker device, as the more contemporary Aveir leadless pacemaker device was not approved by FDA for commercial use during that time.

## Conclusion

The real-world prevalence of pericardial effusion requiring percutaneous or surgical-based intervention after leadless pacemaker implantation was 1.1% in the United States, which was lower than what was demonstrated in the controlled trials. Advanced age, female sex, chronic lung disease, chronic kidney disease, and connective tissue +disorders were associated with a higher likelihood of a serious pericardial effusion. Pericardial effusion requiring intervention was independently associated with mortality, prolonged length of stay, and increased hospitalization costs after leadless pacemaker implantation.
